# Whole‐genome sequencing revealed an interstitial deletion encompassing *OCRL* and *SMARCA1* gene in a patient with Lowe syndrome

**DOI:** 10.1002/mgg3.876

**Published:** 2019-08-03

**Authors:** Bixia Zheng, Qiuxia Chen, Chunli Wang, Wei Zhou, Ying Chen, Guixia Ding, Zhanjun Jia, Aihua Zhang, SongMing Huang

**Affiliations:** ^1^ Nanjing Key Laboratory of Pediatrics Children’s Hospital of Nanjing Medical University Nanjing China; ^2^ Department of Nephrology Children’s Hospital of Nanjing Medical University Nanjing China; ^3^ Jiangsu Key Laboratory of Pediatrics Nanjing Medical University Nanjing China

**Keywords:** CNVseq, copy number variation, Lowe syndrome, *OCRL* gene

## Abstract

**Background:**

Lowe syndrome is a rare X‑linked syndrome that is characterized by involvement of the eyes, central nervous system, and kidneys. The aim of the present study was to determine the molecular basis of four patients with congenital cataract, infantile congenital hypotonia, and proximal renal tubular defect.

**Methods:**

Four children who met the clinical manifestations of Lowe syndrome were enrolled in this study. Patients’ clinical information on eyes, central nervous system, kidneys, and family histories, etc., were reviewed and analyzed. After obtaining informed consent, we performed a mutation analysis of *OCRL* gene using direct sequencing. Because of failure of PCR amplification, low coverage shortread whole genome sequencing (CNVseq) analysis was performed on one proband. Real‐time PCR was subsequently performed to confirm the CNV that was detected from the CNVseq results.

**Results:**

We identified three *OCRL* allelic variants, including two novel missense mutations (c.1423C>T/p.Pro475Ser, c.1502T>G/p.Ile501Ser) and one recurrent nonsense mutation (c.2464C>T/p.Arg822Ter). Various bioinformatic tools revealed scores associated with potential pathogenic effects for the two missense variants, and protein alignments revealed that both variants affected an amino acid highly conserved among species. Since deletion of the entire gene was suspected in a patient, CNVseq was used, identifying an interstitial deletion to approximately 190 kb, encompassing *OCRL*, and *SMARCA1* gene. Moreover, the hemizygous CNV was confirmed by qPCR. Reviewing another case reported in the literature, we found that the deletion of OCRL and nearby genes may contribute to a more severe phenotype and premature death.

**Conclusions:**

This is the first report of an interstitial deletion encompassing *OCRL* and *SMARCA1* gene in Lowe syndrome. Our results expand the spectrum of mutations of the *OCRL* gene in Chinese population. Moreover, whole‐genome sequencing presents a comprehensive and reliable approach for detecting genomic copy number variation in patients or carriers in the family with rare inherited disorders.

## 1 INTRODUCTION

Lowe syndrome (OMIM, #309000) is a multisystem disorder characterized by ocular abnormalities, neurological involvement, and renal dysfunction of the Fanconi type (Charnas, Bernardini, Rader, Hoeg, & Gahl, 1991). In 1992, the causative gene *OCRL* (OMIM, *300535) was identified on the X chromosome, which encodes a 5‐phosphatase that acts preferentially on phosphatidylinositol 4,5‑bisphosphate (PI(4,5)P2) (Attree et al., 1992). Mutations in *OCRL* also cause Dent disease 2 (OMIM, #300555), a milder phenotype that only results in proximal renal tubulopathy (Hoopes et al., 2005).

*OCRL* is composed of 23 exons. Exons 2–5 encode the pleckstrin homology (PH)‐like domain, exons 9–15 encode the 5‐phosphatase catalytic domain, and exons 16–22 encode the ASPM, SPD‐2, Hydin (ASH) and RhoGAP‐like domains of the *OCRL* protein (Faucherre et al., 2003; Mao et al., 2009; Peck, Douglas, Wu, & Burbelo, 2002). Since this time, more than 260 different nonsense, frameshift, splice site, or missense mutations have been annotated for *OCRL* gene in the Human Gene Mutation Database (http://www.hgmd.cf.ac.uk), and they are distributed across all exons. Of note, nearly all mutations associated with Lowe syndrome are mapped in exons 8–23, which comprises the inositol polyphosphate 5‑phosphatase, ASH and RhoGAP‐like domains, whereas the majority of mutations that cause Dent disease 2 are located in exons 1–7, which encompass the PH domain.

Differing clinical presentations have been noted in unrelated individuals with the same *OCRL* pathogenic variant. The severity of the clinical phenotype of patients with Lowe syndrome and Dent disease 2 can vary considerably, even between patients with mutations that are predicted to cause complete loss of function of the protein or between patients who share the same mutation, suggesting that genetic background might influence the clinical expression of the disease (Hichri et al., 2011; Recker et al., 2015). In this study, we performed a detailed genetic and phenotypic analysis of four patients presenting with Lowe syndrome. In particular, low coverage shortread whole genome sequencing (CNVseq) identified an interstitial deletion encompassing *OCRL* and *SMARCA1* gene.

## 2 MATERIALS AND METHODS

Four male boys who presented with congenital cataract, infantile congenital hypotonia, and proximal renal tubular defect were included in the study. Genomic DNA was isolated from peripheral leukocytes of the proband and their parents using a DNA isolation kit (Tiangen, China) according to the manufacturer's protocol.

All patients and families gave informed consent for participation in the study. The study protocol was approved by the ethics committee of Children's Hospital of Nanjing Medical University.

### 2.1 Mutation analysis by Sanger sequencing

All exonic sequences and intron–exon boundaries of *OCRL* (Ensembl Accession ENSG00000122126) were amplified by polymerase chain reaction (PCR) using primers designed by Primer Premier 5.0. All primers are listed in Supplemental Table S1. The PCR products were gel‐ and column‐purified and directly sequenced. The purified PCR fragments were then sequenced using Big Dye Terminator (Applied Biosystems) on an ABI 3130 genetic analyzer (Applied Biosystems). All variants were denoted based on the NCBI reference sequence for *OCRL* (NM_000276.3).

### 2.2 Copy number variant (CNV) analysis by WGS

Approximately 1 μg of DNA was used to prepare library. Genomic DNA sample was sheared to 200–300 bp by sonication and all other steps were followed according to the manufacturer's protocol. Subsequently, the DNA was sequenced on the Illumina NovaSeq6000 platform.

Raw image files were processed by the BclToFastq（Illumina）for base calling and generating the raw data. After removing reads with adaptors and low quality reads, the clean data was aligned to the NCBI human reference genome (hg19) using BWA. 100 kb and above CNV can be detected using chigene independently developed CNV analysis software (Chigene Translational Medicine Research Center). Decipher, ClinVar, OMIM, DGV, and ClinGen databases were used to analyze the genes included in CNV and other disease‐related annotations in order to obsess the Pathogenicity classification of CNV.

### 2.3 qPCR confirmation of the CNV

The primer pair sequences are shown in Supplementary Table S1. Samples for qPCR were assayed in triplicate using the Takara SYBR Green with GAPDH genomic content used as an endogenous control for normalization of the data. The ΔΔCt comparison method was used to measure relative DNA content on the QuantStudio™ 3 instrument (ThermoFisher).

### 2.4 Bioinformatics predictions

The function effects of the novel missense mutations identified in this study were predicted with the software PolyPhen‐2 (http://genetics.bwh.harvard.edu/pph2/), SIFT (http://sift.jcvi.org), and mutationtaster (http://www.mutationtaster.org/).

## 3 RESULTS

### 3.1 Clinical presentation

Clinical features of our patients are listed in Table 1. The mean age of the patients at diagnosis was 17.5 months (range, 6 months 15 days–2 years 4 months).

**Table 1:** Clinical and genetic characterization of patients examined for *OCRL* gene mutations

Subject	Patient 1	Patient 2	Patient 3	Patient 4
Gender	Male	Male	Male	Male
Age	2 years 1 month	2 years 4 month	6 month 15 days	11 months
Mutation	c.1423C>T	c.2464C>T	interstitial deletion	c.1502T>G
Protein change	p.Pro475Ser	p. Arg822Ter	—	p. Ile501Ser
Neurological symptoms	Severe hypotonia, intellectual impairment	Severe hypotonia, intellectual impairment	Severe hypotonia, hypermyotonia and amyotrophy	Severe hypotonia
Ocular manifestations	Strabismus,nystagmus, congenital cataract	Congenital cataract	Congenital cataract	Congenital cataract,nystagmus
Renal manifestations	Proteinuria, hypercalciuria	Proteinuria, hypercalciuria, metabolic acidosis	Proteinuria, hypercalciuria, metabolic acidosis,nephrocalcinosis	Proteinuria, hypercalciuria, metabolic acidosis,nephrocalcinosis
Rickets	Square head	Pigeon chest, mild bracelet sign	—	—
Other features	Growth retardation	Growth retardation, right hydrocele of testis	Growth retardation, anemia, poor brain development, feeding difficulties	Growth retardation, ventricular septal defect,cryptorchidism

Note

Accession no: NM_000276.3.

#### 3.1.1 Eyes involvements

Bilateral cataracts were observed at birth, and all of them had accepted early cataract extraction. Strabismus was also presented in patient 1 and patient 4. The mother of patient 3 and patient 4 had cataract and strabismus, respectively.

#### 3.1.2 Neurological symptoms

Severe hypotonia at birth with loss of deep‐tendon reflexes were observed in all of patients. Patient 1 and 2 have intellectual impairment. Hypermyotonia and amyotrophy occurred in patient 3. Besides, patient 3 also has poor brain development, and the brain MRI shows that the extra‐brain space of the bilateral frontal sac is widened, the corpus callosum is thin, and the lateral ventricle is abnormal. He died at 7 months of age from unknown causes.

#### 3.1.3 Renal manifestations

Proteinuria developed in the first months of life in all affected patients, and urinary α1 microglobulin was elevated. Hypercalciuria was observed in all of them, and nephrocalcinosis was found in patient 3 and patient 4. Three of them also showed metabolic acidosis. The eGFR at the last follow‐up was normal in all patients.

#### 3.1.4 Other presentations

Growth retardation was observed in all patients. In two patients (patient 1 and patient 2), rickets were observed, such as square head, pigeon chest, mild bracelet sign. Anemia was observed in two patients (patient 3 and patient 4). Patient 3 also showed platelet dysfunction but without bleeding. Hydrocele testis and cryptorchidism were observed in patient 2 and 4, respectively.

### 3.2 Identification of three‐point mutations in *OCRL* gene

**Figure 1:**
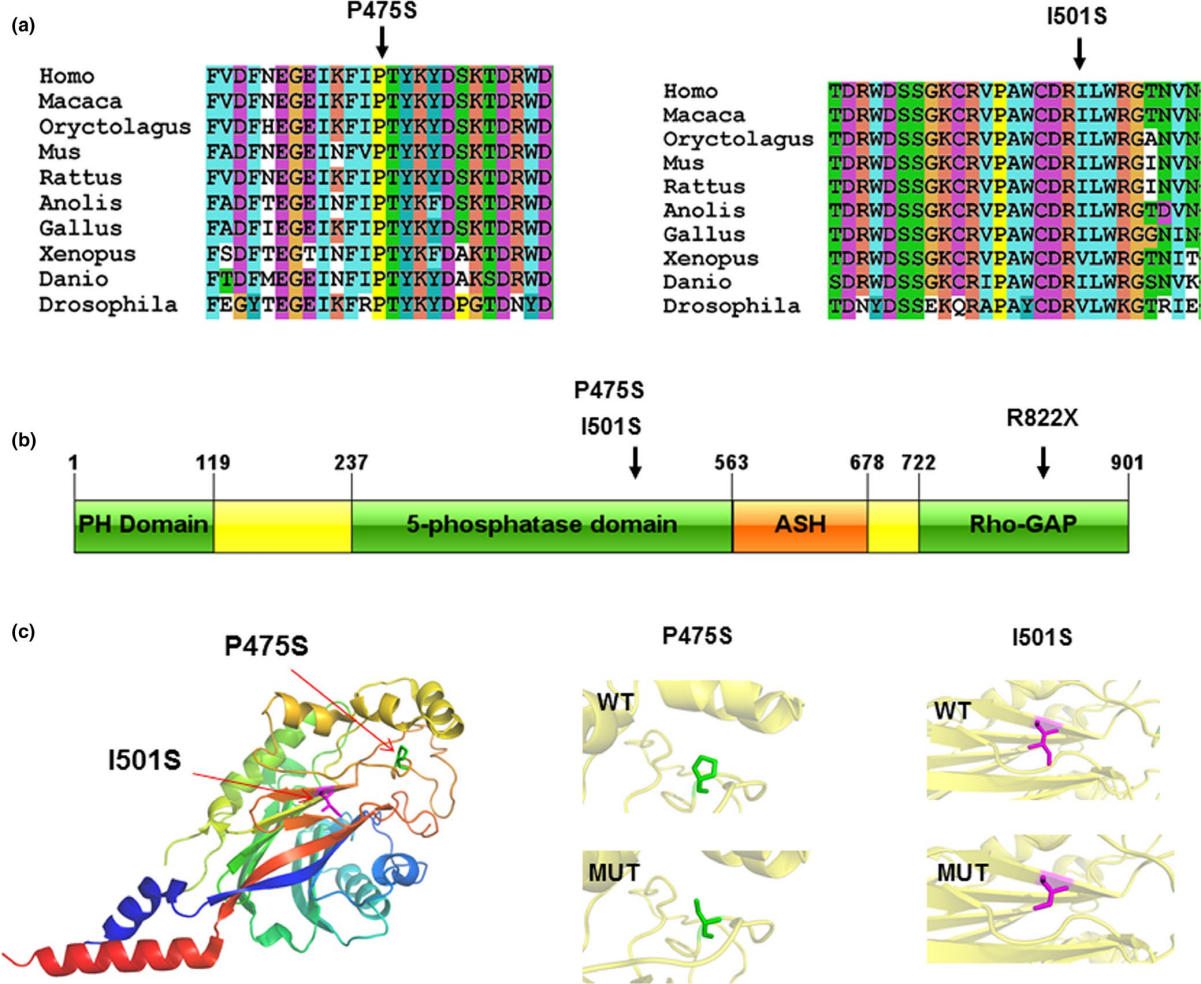
(a) Alignment of *OCRL* orthologs in different species around the mutated amino acids residues; (b) Schematic illustration showing the distribution of identified alterations relative to the OCRL protein structure depicting the pleckstrin homology (PH)‐like domain, 5‐phosphatase catalytic domain, ASH and RhoGAP‐like domains. (c) Structural visualization of the identified *OCRL* missense alterations using the crystal structure of OCRL in complex with a phosphate ion (PDB accession 4CMN). The residues present at our two mutation sites are shown as sticks

By direct sequencing of the *OCRL* gene, we identified three hemizygous point mutations (c.1423C>T/p.Pro475Ser, c.1502T>G/p.Ile501Ser, and c.2464C>T/p.Arg822Ter). All the three mutations were maternally inherited. The two missense mutations have not been reported in the genomic databases or the literature at the time of query. Various bioinformatic tools revealed scores associated with potential pathogenic effects for the two missense variants. In addition, protein alignments revealed that both variants, located in the 5‑phosphatase domain of the protein, affected an amino acid highly conserved among species (Figure 1a). The nonsense mutation p.Arg822Ter, that has been previously described, was detected in patient 2. The premature stop codon at residue 822 leads to the partial loss of RhoGAP‐like domain, which is characterized by the presence of many sites that mediate interaction of *OCRL* with RabGTPases and proteins associated with actin polymerization.

### 3.3 Identification and validation of a hemizygous deletion in Xq25‐26

**Figure 2:**
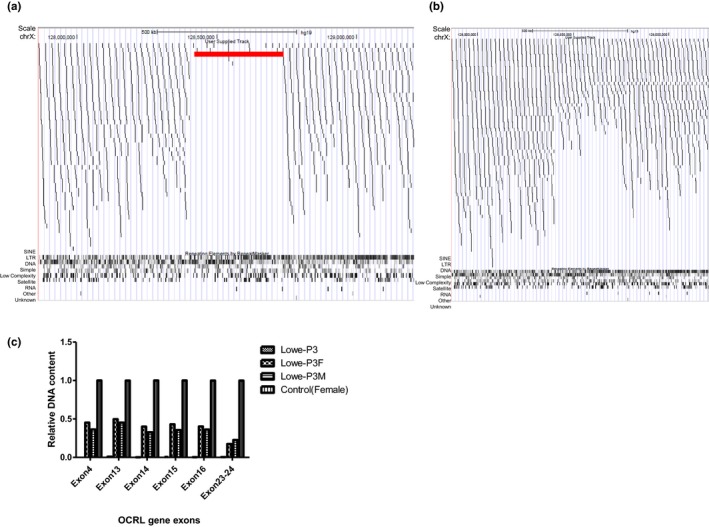
The detection of a 190kb deletion on Xq25‐26. Datapoints for the low coverage whole genome sequencing tracks represent the genomic start coordinate of each aligned sequence read. (a) Datapoints from the affect proband. The red track displays the 190 kb deletion. (b) Datapoints from the heterozygous mother. (c) qPCR analysis of relative DNA content in whole blood from the control subject, father, mother, and proband

Deletion of the entire gene was suspected in patient 3 because of failure of PCR amplification. Copy number variant analysis of the WGS data identified an interstitial deletion encompassing the *OCRL* and *SMARCA1* gene (chrX: 128548236 ‐128738236, q25‐q26.1) in the proband (Figure 2a). The deletion was heterozygous in his mother. We designed several primers in the *OCRL* gene to confirm the deletion. Validation of this deletion by qPCR confir hemizygous and heterozygous in the proband and his mother, respectively (Figure 2b).

## 4 DISCUSSION

We identified four variants of the *OCRL* gene in four patients with Lowe syndrome, including two novel missense mutations, one recurrent nonsense mutation and a microdeletion encompassing *OCRL* and *SMARCA1* gene. The clinical features present in these patients are generally consistent with the pathophysiology found in Lowe syndrome, including: bilateral dense congenital cataracts, infantile congenital hypotonia, delayed development, LMW proteinuria, metabolic acidosis, rickets, hypercalciuria, and nephrocalcinosis. Additionally, other urogenital abnormalities including cryptorchidism and hydrocele testis also were observed in our patients. All the diagnosis of our patients was delayed. Female heterozygotes with Lowe syndrome can have mild features of the disorder. The mother of patient 3 and patient 4 had cataract and strabismus, respectively. In addition, the mother of patient 4 had intellectual disability. The life expectancy of patients with Lowe syndrome is estimated to be approximately 40 years and death is usually secondary to CKD and its related complications (Zaniew et al., 2018). Patient 3 died at 7 months of age from unknown causes.

Copy number variations (CNVs) within the human genome have been linked to a diversity of inherited diseases and phenotypic traits. It is estimated that up to 10% of the human genome is affected by CNVs (Truty et al., 2018). According to HGMD, there were 11 gross deletion of *OCRL* gene have been reported. Here, we identified an interstitial deletion encompassing the *OCRL* and *SMARCA1* gene in an affected male with severe features of Lowe syndrome. Only one patient carrying the deletion of the *OCRL* and nearby genes has been described. Addis et al identified a genomic deletion in Xq25 covering the entire *OCRL* gene and three other genes (*SMARCA1*, WDR4OB, and ACTRT1) in an Italian patient who died at 5 months of age from unknown causes (Addis et al., 2007). Similarly, in our study, the patient harboring the interstitial deletion encompassing *OCRL* and *SMARCA1* gene presented with severe features of Lowe syndrome after birth and died at 7 months of age from unknown causes. In particular, he had poor brain development. The *SMARCA1* gene in the deleted interval does not have known phenotypic or disease variations. *SMARCA1* gene, also known as SNF2L, was expressed in terminally differentiated neurons after birth and in adult mice, as well as in adult ovaries and testes (Lazzaro & Picketts, 2001). *SMARCA1* was found to be a component of a second chromatin remodeling complex, called CERF that contains the CECR2 protein, a transcription factor involved in neurulation and a cause of exencephaly in mice when mutated (Banting et al., 2005). In addition, previous study also demonstrated that SNF2L has a genetic link with Foxg1, which play a key role in the regulation of neurogenesis (Yip et al., 2012). Therefore, it could not be excluded the possibility that additional features caused by the deletion of *SMARCA1* aggravate the phenotype of Lowe syndrome.

Array comparative genomic hybridization (aCGH) is the common method of choice for performing genome‐wide screening for large genomics abnormalities (McMullan et al., 2009; Miller et al., 2010). Most of reported large gene deletions in *OCRL* gene were identified by aCGH or FISH (Addis et al., 2007; Peverall, Edkins, Goldblatt, & Murch, 2000). More recently, though, the use of next‐generation sequencing (NGS) methods for the detection of copy number variants has also been increasing (Gross et al., 2018; Wood et al., 2010). In particular, CNVseq is a reliable and cheap approach which has diagnostic sensitivity for germline CNVs comparable to that of standard aCGH reagents (Zhou et al., 2018). Here, we showed that CNVseq offers an alternative data source to allow for the detection and characterization of the copy number across different genomic regions in a single experiment.

This is the first report of an interstitial deletion encompassing *OCRL* and *SMARCA1* gene in Lowe syndrome. The findings related to the spectrum of large genomic deletion and some new point mutations will increase our understanding on the genetic characteristics of Lowe syndrome in Chinese population. Moreover, the use of the whole‐genome sequencing technique facilitated carrier and prenatal testing for the deletion in the family with Lowe syndrome.

## CONFLICT OF INTEREST

The authors have no conflicts of interest to declare.
