# Supplementary material for: Whole‐genome sequencing revealed an interstitial deletion encompassing OCRL and SMARCA1 gene in a patient with Lowe syndrome
Source: Mol Genet Genomic Med. 2019 Aug 3;7(9):e876. doi: 10.1002/mgg3.876 (PMC6732312; doi:10.1002/mgg3.876)
Supplement: Supplementary file 1 [file MGG3-7-e876-s001.doc]

| Exons | Sequence |
| --- | --- |
| OCRL-E4-Forward | 5’-GAGGAGTTCCATTTGGTTACTTC-3’ |
| OCRL-E4-Reverse | 5’-CAGCAATGATTCAACTGACAGC-3’ |
| OCRL-E13-Forward | 5’-GTAGTTGCCCTCAAAGGATTAG-3’ |
| OCRL-E13-Reverse | 5’-GGGAGTGATGGAAACGTCTTAC-3’ |
| OCRL-E14-Forward | 5’-CTTTGAAATAGGAACAGTGGC-3’ |
| OCRL-E14-Reverse | 5’-GGCACTGAGCCATTAGGTTTAT-3’ |
| OCRL-E15-Forward | 5’-GAGCTTGCAAAATAGTAGGGA-3’ |
| OCRL-E15-Reverse | 5’-CACTAAAAGGCCTGGAGCATT-3’ |
| OCRL-E16-Forward | 5’-GGATGTTGTTTGCACCACAG-3’ |
| OCRL-E16-Reverse | 5’-CTACTAAACAGCCGTGCTAC-3’ |
| OCRL-E23-24-Forward | 5’-CAGATGAAATGGGTCCTGC-3’ |
| OCRL-E23-24-Reverse | 5’-AAAGGAGGGATTAGGAAACG-3’ |

Supplementary Table S1 The primer pair sequences for qPCR
